# The influence of cholesterol on the 5-year all-cause mortality of Parkinson patients with or without deep brain stimulation

**DOI:** 10.1177/00368504261463435

**Published:** 2026-07-22

**Authors:** Gabriel C. Roth, Marco Treven, Christof Brücke, Johann Lehrner

**Affiliations:** 1 Department of Neurology, 27271 Medical University of Vienna, Vienna, Austria

**Keywords:** Parkinson disease, 5-year all cause mortality, deep brain stimulation, cholesterol, neuropsychological battery

## Abstract

**Objective:**

Parkinson’s disease (PD) is a progressive neurodegenerative disorder marked by motor and non-motor symptoms. While cholesterol metabolism has been implicated in PD pathogenesis, its role in long-term mortality remains unclear. The intention of this study was to evaluate if cholesterol parameters impact the 5-year all-cause mortality of PD patients.

**Methods:**

This study has a retrospective cross-sectional design, including a total of 212 PD, patients which were grouped by treatment (DBS or BMT). Cognitive function, depressive symptoms, and motor status were assessed with standardized instruments. Cholesterol parameters (total cholesterol, LDL, HDL, TC/HDL ratio) were measured within ± 90 days of neuropsychological testing. Cox proportional hazards models and Kaplan-Meier analyses were used to examine associations with 5-year mortality.

**Results:**

Five-year survival likelihood was 88.4% for DBS and 66.7% for BMT. However, cholesterol parameters emerged as non-significant predictors of mortality in the Cox regression model. Age occurred as the only significant predictor.

**Conclusion:**

Cholesterol levels do not appear to predict 5-year mortality in PD patients. Age was the strongest risk factor. Further studies are recommended to clarify cholesterol’s role in PD progression and mortality.

## Introduction

Parkinson’s disease (PD), the second most common neurodegenerative disease after Alzheimer’s disease (AD), affects around 0.3% of the population of industrial countries.^1^ The disease has a variety of symptoms appearing at different stages, which can be divided into two categories: motor symptoms and non-motor symptoms. Common motor symptoms include bradykinesia, rigidity, rest tremor and postural instability. Common non-motor symptoms include anosmia, gastrointestinal problems, depression and cognitive decline to name just a few.^2^ PD is an incurable disease, but its symptoms can be treated medically or surgically with deep brain stimulation (DBS).^3^ DBS has been show to be more effective in relieving motor symptoms than medical therapy, with DBS patients having a significantly better improvement in their Hoehn & Yahr stage (H&Y-S) and their MDS UPDRS Motor III - scale.^4^

Recent studies found that cholesterol metabolites, also called oxysterols, in particular 24-S-Hydroxycholesterol (24-S-HC) and 27-Hydroxycholesterol might be involved in the development and progression of PD. The oxysterol 24-S-HC may effect neurotoxic effects by inducing neuronal cell death while 27-HC was linked to the spread of alpha-synuclein pathology in an animal model.^5,6^ In PD patients a significant correlation was observed between 24-S-HC levels in cerebrospinal fluid (CSF) and disease duration.^7^ Furthermore, 24-S-HC was associated with UPDRS I scores at one year follow-up, further suggesting a potential link with disease progression.^8^ It has also been observed that plasma cholesterol levels tend to be lower in PD patients when compared with healthy controls^9^ and that there is an inverse relationship between cholesterol levels and the risk of developing PD.^9,10^ These findings suggest that cholesterol parameters may not only have a potential role in PD pathophysiology but may also be associated with clinical outcomes in PD patients including mortality.

Although Alzheimer’s disease (AD) and PD are distinct diseases, they share important characteristics such as both being a progressive neurodegenerative disease in which demographic factors, cognition and mental state influence long-term outcomes. Studies in AD, consistently showed that factors such as age, male gender, neuropsychological testing performance have a relevant influence on the five-year mortality of AD. With the exception of Gerschmann and Lehrner (2024), depressive symptoms were also identified as a risk factor. Homocysteine and Hemoglobin A1c levels were also significant risk factors while Triiodothyronine levels showed no significant influence.^11–14^

Based on this evidence, the primary aim of this current study was to investigate whether blood cholesterol parameters are associated with 5-year all-cause mortality in PD patients. Furthermore, we explored if this association remains significant after adjusting for demographic, clinical and neurocognitive performance variables.

## Materials and methods

This study is a retrospective cohort study with a cross-sectional, single center design. Subjects included in this study were all patients of the outpatient clinic of the Department of Neurology, Medical University of Vienna. The data is part of an ongoing study at the Medical University of Vienna registered with the ethics vote EK 1159/2023 from the ethics committee of the university. The present sample is partially overlapping with a previously published cohort which investigated the influence of depressive symptoms on 5-year all-cause mortality of PD patients.^15^ The current study differs in its primary objective investigating the influence of blood cholesterol levels. A positive ethics vote by the ethics committee of the Medical University of Vienna has been received on the 10.12.2024 and is registered under the number EK 2318/2024 and was performed in accordance with the Declaration of Helsinki of 1975 as revised in 2024. The subjects were recruited in the period of Feb. 1999 – Oct. 2024. The follow-up period was calculated using the date of death and the date of examination. Date of death information was available until December 2023 via Statistik Austria and the Allgemeines Krankenhaus Informations System (AKIM). Patients without a recorded death event were treated as censored cases and individual survival times were calculated using the last documented contact until December 2024. Signed consent from the patients was collected for the ongoing study from which the data of this study stems. For this study no signed consent was collected as it was a retrospective data analysis with pseudoanonymized data. The data was extracted from the Research, Documentation and Analysis (RDA) system of the Medical University of Vienna and AKIM system of the Allgemeines Krankenhaus Wien (AKH) by the author Gabriel C. Roth. Data integrity and correct transfer of the data was continuously double-checked. The reporting of this study conforms to STROBE guidelines.^16^ A summary of all abbreviations used in this publications is presented in Table 1. Various measures were taken to minimize potential bias, including the use of predefined inclusion criteria, standardized clinical, neuropsychological, and laboratory procedures and continuous monitoring of data extraction and transfer.

**Table 1.** Abbreviations.

Abbreviation	Description
AD	Alzheimer’s disease
AKH	Allgemeines Krankenhaus Wien
AKIM	Allgemeines Krankenhaus Informationsmanagement
BMT	Best medical treatment
CSF	Cerebrospinal fluid
DBS	Deep Brain Stimulation
EK	Ethikkommission
GDS-15	Geriatric Depression Scale 15
HDL (-C)	High density lipoprotein (-cholesterol)
H&Y-S	Hoehn and Yahr - Scale
KM	Kaplan-Meier
KMO	Kaiser-Meyer-Olkin
LDL (-C)	Low density lipoprotein (-cholesterol)
MCI	Mild cognitive impairment
MDSTF	Movement Disorder Society Task Force
MDS-UPDRS	Movement Disorder Society - United Parkinson Disease society rating scale
MMSE	Mini mental state examination
NTBV-15	Neuropsychological Test Battery Vienna-15
PCA	Principal component analysis
PD	Parkinsons disease
RDA	Research, Documentation and Analysis
STROBE	Strengthening the Reporting of Observational Studies in Epidemiology
TC	Total cholesterol
UPDRS	United Parkinson Disease society rating scale
VNTB	Vienna neuropsychologische Testbatterie
VVT 3.0-SCO	Vienna Visuoconstruction Test 3.0 screening copy
24-(S)-HC	24-(S)-Hydroxycholesterol
27-HC	27-Hydroxycholesterol

### Participants

As this is a retrospective data analysis, patients were not recruited for this study but for the ongoing study mentioned in the methods section using the inclusion criteria mentioned. Only exclusion criteria were applied to the patients retrospectively to be included in the analysis.

*Inclusion criteria*: Patients needed to meet the UK Parkinson’s Disease Society Brain Bank Criteria for PD,^17^ DBS patients needed to have suffered from PD for at least 5 years without a positive response to L-DOPA treatment and motor complications non-responsive to drug treatment or a treatment resistant tremor, patients without DBS had to receive the best medical therapy (BMT) available for PD.

*Exclusion criteria*: Secondary Parkinsonism or degenerative process with symptoms similar to Parkinson, severe cognitive impairments e.g. dementia and uncontrolled psychiatric disorders not including depression, missing data on cholesterol parameters and or mortality data. Cholesterol data outside of ± 90days of the neuropsychological testing.

The original patient collective consisted of N = 419 patients. After application of the inclusion and exclusion criteria n = 81 patients had to be excluded because of missing cholesterol parameters, n = 10 patients had to be excluded because the date of death was outside of the observational period, n = 107 patients were excluded because the cholesterol data was outside of the ± 90d range, n = 9 patients were additionally excluded because of partially missing cholesterol data finally concluding with a sample size of n = 212 protocols which could be considered in this study.

### Instruments

Cognitive and mental status of the participants was assessed using the Mini Mental State Examination (MMSE),^18^ Vienna Visuoconstructive and Visual Memory Test (VVVMT),^19,20^ Geratric Depression Scale 15 (GDS-15),^21^ Neuropsychological Test Battery Vienna 15 (NTBV-15).^22,23^ The NTBV-15 and VVT 3.0-SCO are available for download from www.psimistri.com. The MMSE is a screening test for dementia/cognitive impairment with a maximum of 30 points, it was not intended to be used as a clinical diagnostic tool and should not replace a full clinical examination.^18^ In the context of this study the MMSE was used to screen for dementia and exclude affected patients. The GDS-15 inventory is short version of the original 30 item scale used to screen for depression in elderly persons. The score ranges from 0 – 15 with 0 - 4 points indicationg a normal state of mind, 5 - 9 points mild depression and 10 - 15 points moderate to severe depression.^21^ The VVVMT-SVCis a neuropsychological test to assess visuoconstructive function of patients consisting of three figures (a clock, two overlapping pentagons and cube) that have to be copied by the patient. The patients are then awarded between 0 - 10 points.^20^ The NTBV-15 represents a short version of the NTBV which was originally introduced as the “Vienna Neuropsychologische Testbatterie (VNTB)” in 2007 it assesses cognitive function in multiple different neuropsychological domains including psychomotor speed, attention, language, memory and executive function.^22,23^ There is currently no directly recommended gold standard neuropsychological assessment but several recommendations of different neuropsychological test batteries.^24^ The MDS itself recommends that the neuropsychological test batteries include the domains attention and working memory, executive function, language, memory and visuospatial function.^25^ The VVVMT-SVC and NTBV-15 used for this study largely cover the recommended domains.

The motor symptoms of the patients were evaluated using the Hoehn & Yahr scale (H&Y-S)^26^ and the MDS-UPDRS-Motor III score.^27^ The H&Y- S is a five stage scale used to rating the extent of patient disability before and after treatment and monitoring disease progression. In the first stage patients are affected unilaterally and have minimal disability or no functional affection at all. In the fifth and last stage patients are confined to their bed or wheelchair if no outside aid is available.^26^ The MDS-UPDRS-Motor III score is a part of the larger MDS-UPDRS scale which itself is a modified version of the UPDRS from 1980. The MDS-UPDRS has a total of four parts which evaluate non motor and motor symptoms impact on daily living, motor symptoms generally and motor complications. The motor examination part of the scale, used in this study, consists of 30 items with a score range of 0 - 132.^27^ The MDS-UPDRS-scale represents the current gold standard for rating disease progression in Parkinson patients.^28^

Blood sampling for the cholesterol was done at one of the patients’ visits at the Department of Neurology, Medical University of Vienna and tested by the Department of Laboratory Medicine, Medical University of Vienna. Blood samples had to be within ± 90 days of the neuropsychological testing to be viable for statistical analysis. The cholesterol parameters analyzed for this study were total cholesterol (TC), low density lipoprotein (HDL), high density lipoprotein (HDL) and the TC/HDL-ratio. The reference values for TC, HDL and LDL and the analysis methods used for blood sampling are available at the website of the General Hospital of Vienna (AKH-Wien).^29^

### Statistical analysis

The descriptive and inductive statistics were conducted using the software IBM SPSS^®^ 30.0 (IBM Corp., Armonk, NY) for Windows^®^ 11. In the context of inferential analyses the alpha level was set at 5% corresponding to the type-I error, so that a result with *p* ≤ 0.05 is interpreted as significant in the context of inferential statistics. To assess the practical significance and relevance of inferential results the corresponding effect sizes *r* in the context of U-testing and *d* in the context of unpaired t-testing according to Cohen’s classification were applied. Value ranges for the effect size *r* are defined as ≥ .10 as small, ≥ .30 as moderate and ≥ .50 as substantial and for the effect size *d* as ≥ 0.20 as small, ≥ 0.50 as moderate and ≥ 0.80 as substantial.

The analyses were based on a comparative evaluation of the two treatment subgroups BMT and DBS. In all calculations the per protocol approach was used and no imputations were carried out except the principal component analysis of the NTBV subtests, where 2.45% missing values had to be imputed by the mean substitution method. For the descriptive statistics we calculated mean ± *SD* in addition with the minimum and maximum. In case of skewedly distributed data, median and interquartile ranges Q1 25% and Q3 75% were displayed. Distributions were evaluated using the Kolmogorov-Smirnov and Shapiro-Wilk test. Intergroup comparisons were done using t-tests or U-testing depending on data distribution.

To reduce the redundancy of the NTBV-15 subtests and achieve a dimensional overview a principal component analysis (PCA) with subsequent orthogonal Varimax-rotation according to Kaiser’s normalization was carried out. The benefits of this approach are independent z-standardized factor-scores improving comparability and interpretability of extracted neuropsychological dimensions between subgroups and substituting missing values.

To test if there is an association between cholesterol parameters and mortality a Cox proportional hazards model was used. Different covariates and potential predictors including all cholesterol parameters (TC, HDL, LDL, TC/HDL), age, sex, educational years, GDS-15 (representative for depression), NTBV-factor scores (representative for cognitive performance) and the treatment subgroups were added into the model as hierarchical blocks. The first block consisted of only the cholesterol parameters, in the second block we added the parameters age and sex, in the third block educational years were added, in the fourth block GDS-15 scores, the fifth block NTBV-factor scores and in the sixth and final block containing all of the parameters the treatment modality was added. A Kaplan-Meier (KM) - survival function was calculated for the 5-year survival of the two treatment subgroups. To compare the survival rates of the subgroups a log-rank (Mantel-Cox) procedure was used. Finally, a bivariate scatterplot for the relation between TC and age considering the two PD treatment groups with according regression equations was generated.

## Results

Demographic, clinical and cholesterol parameters are listed and summarized in Table 2. Regarding the effect sizes, it should be noted that a negative prefix indicates a lower value for the DBS subgroup. There were significant differences between the two subgroups regarding age *p* = .001, age at onset *p* = .001 and disease duration *p* = .001. The proportion of male patients in the study collective was 60,8%, 95%-CI [54.2%; 67.4%]. An intergroup comparison revealed no significant difference for sex distribution *p* = .875 but when looking at the whole study population there are significantly more male patients *p* = .002. For neurocognitive test performances there are significant differences between the treatment groups regarding MMSE *p* = .001, VVVMT-SVC 3.0 *p* = .003 and GDS-15 *p* = .017. Disease severity H&Y-S and motor symptoms severity MDS-UPDRS Motor III both showed significant differences with p = .001. Regarding cholesterol parameters only the TC/HDL ratio showed a significant difference with *p* = .028.

**Table 2.** Key values (M ± SD, Mdn (Q1-Q3)) of metric parameters with significance assessment and effect sizes r and d; frequencies (n) and proportions (column percentages) considering PD-subgroups.

Parameter	BMT (n=134)	DBS (n=78)	Total (N=212)	*p*-value effect size
age (year)	134	78	212	​
*M* ± *SD*	69.5 ± 10.0	59.7 ± 8.0	65.9 ± 10.5	<.001^**^
*Mdn* (IQR)	70.8 (63.6; 77.1)	60.8 (55.9; 65.7)	66.1 (58.9; 73.1)	*r* = -.47
age at onset (year)	109	75	184	​
*M* ± *SD*	61.2 ± 11.4	48.5 ± 8.5	56.1 ± 12.0	<.001^**^
*Mdn* (IQR)	63 (53; 69)	48 (43; 54)	55 (48; 65)	*r* = -.53
disease duration (months)	107	75	182	​
*M* ± *SD*	89.8 ± 62.2	123.0 ± 53.6	103.5 ± 60.9	<.001^**^
*Mdn* (IQR)	75 (44; 132.5)	119 (84; 155)	96 (60; 144)	*r* = +.29
sex	134	78	212	​
female	53 (39,6%)	30 (38,5%)	83 (39,2%)	.875
male	81 (60,4%)	48 (61,5%)	129 (60,8%)	
educational years	133	78	211	​
*M* ± *SD*	10.9 ± 3.6	11.9 ± 4.3	11.3 ± 3.9	.077
*Mdn* (IQR)	10.0 (8; 12)	9.5 (9; 14)	10.0 (8; 13)	*r* = -.12
MMSE (0 – 30)	134	78	212	​
*M* ± *SD*	26.0 ± 2.9	27.7 ± 2.0	26.6 ± 2.7	<.001^**^
*Mdn* (IQR)	27 (25; 28)	28 (27; 29)	27 (25; 29)	*r* = +.32
VVVVMT-SVC (0 – 10)	39	56	95	​
*M* ± *SD*	9.44 ± 1.39	8.79 ± 2.08	9.05 ± 1.85	.003^**^
*Mdn* (IQR)	10 (9; 10)	9 (9; 10)	10 (9; 10)	*r* = -.31
GDS-15 (0 – 15)	110	66	176	​
*M* ± *SD*	4.48 ± 3.41	3.23 ± 2.82	4.01 ± 3.25	.017^*^
*Mdn* (IQR)	4 (2; 7)	2 (1; 5)	3.5 (1; 6)	*r* = -.18
Hoehn & Yahr (staging)	78	51	129	​
1	2 (2,6%)	4 (7,8%)	6 (4,7%)	​
1.5	1 (1,3%)	0	1 (0,8%)	​
2	36 (46,2%)	40 (78,4%)	76 (58,9%)	<.001^**^
2.5	2 (2,6%)	4 (7,8%)	6 (4,7%)	​
3	24 (30,8%)	3 (5,9%)	27 (20,9%)	​
4	13 (16,7%)	0	13 (10,1%)	​
MDS-UPDRS Motor III	88	57	145	​
(0 – 132)	30.1 ± 14.2	20.4 ± 10.1	26.3 ± 13.6	<.001^**^
​	28 (20; 39)	20 (14; 25)	24 (17; 35)	*r* = -.34
Total cholesterol (mg/dl)				
*M* ± *SD*	199.1 ± 41.3	191.6 ± 32.1	196.3 ± 38.3	.146
*Mdn* (IQR)	195.5 (170; 230)	191.0 (166; 213)	194.0 (167.5; 224)	*d* = -0.19
LDL (mg/dl)				
*M* ± *SD*	114.8 ± 35.5	112.3 ± 30.7	113.9 ± 33.7	.584
*Mdn* (IQR)	113.6 (91.8; 114)	106.7 (87; 140.8)	111.6 (89; 140.4)	*d* = -0.08
HDL (mg/dl)				
*M* ± *SD*	60.0 ± 15.8	62.5 ± 16.5	60.9 ± 16.0	.277
*Mdn* (IQR)	57.5 (49; 68)	59.5 (51; 69)	58.0 (50.0; 69.0)	*d* = +0.16
TC/HDL (ratio)				
*M* ± *SD*	3.49 ± .92	3.23 ± .80	3.40 ± .89	.028*
*Mdn* (IQR)	3.40 (2.9; 4.1)	3.25 (2.7; 3.8)	3.30 (2.8; 4.0)	*d* = -0.30

*r* = effect size Pearson’s correlation.

**p ≤ .001.

^*^p ≤ .05; *d* = effect size Cohen’s Δ.

**Figure 1.**
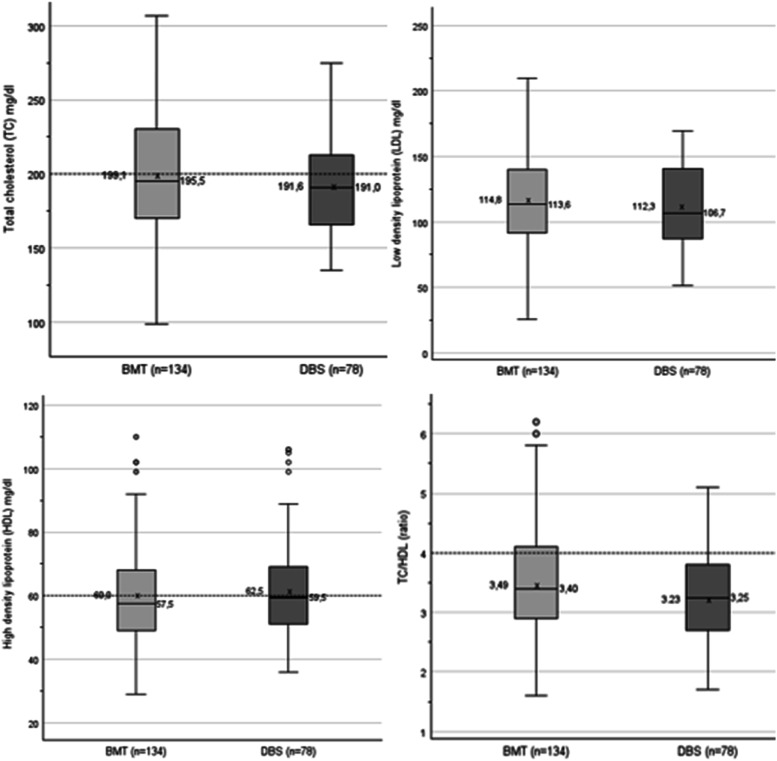
Distribution of different cholesterol parameters regarding PD treatment (with Mdn and position of mean x); upper left total cholesterol, upper right ldl-cholesterol, lower left hdl-cholesterol, lower right TC/HDL-ratio, dotted line representing cut-off levels.

The distributions of the different cholesterol parameters of both groups are visualized in Figure 1. The cutoff values used stem from the AKH laboratory,^29^ since the LDL cutoff depends on cardiovascular risk factors we chose not to include a cutoff. Regarding the calculated value TC/HDL, while there is no general consensus on or recommendation for the cutoff value for a healthy ratio we used the cutoff value of 4.0 for women mentioned in a paper by Millán et al.^30^

Considering the cut-off score ≥ 5 of the GDS-15, 38.6%, 95%-CI [31.4%; 45.8%] could be considered as depressed.

**Figure 2.**
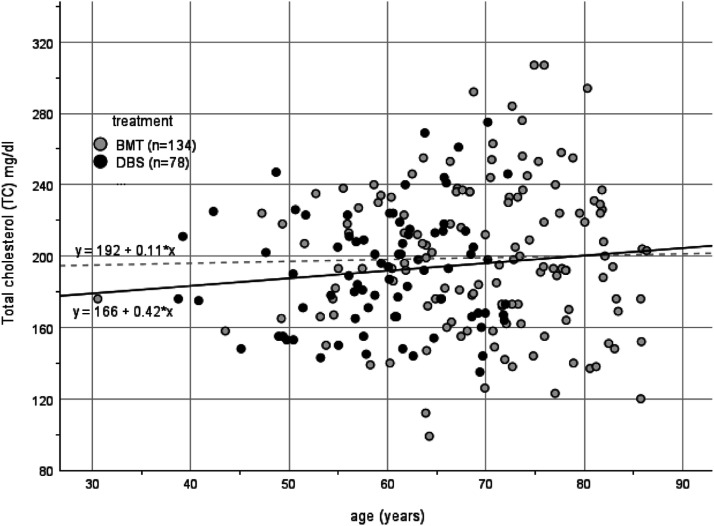
Bivariate scatterplot for the relation between age and total cholesterol with linear regression functions and equations considering the PD treatment subgroups.

Examining the relationship between age and total cholesterol level in PD patients, the correlation coefficient for the entire study collective showed with *r* = .084 (n = 212, *p* = .223 two-tailed) a positive, non-significant increase, as illustrated in the bivariate scatterplot below in Figure 2.

The corresponding regression function for the entire study population was determined as Ŷ_i_ = 176 + 0.308*x_i_ indicating an increase of 3.08 TC mg/dl every decade. The regression equations for the subgroups were as follows BMT: Ŷ_i_ = 192 + 0.105*xi; DBS: Ŷ_i_ = 166 + 0.424*x_i_ indicating an increase of 1.05 TC mg/dl for BMT and 4.24 for DBS every ten years. The associated 95%-CI for the unstandardized slope parameter *B* are overlapping, BMT [-.604; .815] and DBS [-.485; 1.333]. The influence of age on the increase of the TC serum levels was small and showed no significant difference between the two PD subgroups.

A principal component analysis (PCA) was conducted on the NTBV-15 subtests using orthogonal varimax rotation with Kaiser normalization. The rotation converged after 11 iterations. Sampling adequacy was confirmed by a high Kaiser-Meyer-Olkin (KMO) value of 0.874. Of the expected 3,180 values (212 × 15), 2.45% were missing and imputed using mean substitution in SPSS^®^. Subtest loadings (Pearson’s *r*), communalities (h_i_^*2*^), and eigenvalues (λ) were calculated for the extracted components as shown in Table 3.

**Table 3.** Rotated Component Matrix; factor loadings, item communalities and eigenvalue of identified dimensions (n=212).

NTBV-15 subtests	Factor (F), component, dimension				h_i_^2^ (communality)
	F1	F2	F3	F4	
Interference time (c.I.)	**-.807**	-.300	-.156	-.241	.823
Interference total/time (c.I.)	**.806**	.223	.153	.228	.775
AKT concentration I	**-.748**	-.276	-.354	-.090	.769
AKT concentration II	**.719**	.244	.407	-.002	.742
Symbols (c.I.)	**-.667**	-.085	-.429	-.187	.671
Psychomotorioric Speed (TMTA)	**-.616**	-.236	-.365	-.348	.690
VSRT delayed recall	.259	**.818**	.123	.230	.803
VSRT total recall	.376	**.817**	.197	.183	.882
VSRT immediate recall (wordspan)	.229	**.787**	.148	.051	.696
VSRT recognition	.056	**.752**	.104	.148	.601
Maze total/time	.322	.189	**.804**	.117	.801
Maze time	-.349	-.220	**-.794**	-.215	.847
m-BNT naming	.035	.158	.335	**.771**	.733
Animals correct (verbal fluency)	.422	.325	.097	**.663**	.733
PWT-f correct	.514	.152	-.080	**.574**	.623
Eigenvalue λ	4.13	3.10	2.15	1.81	Σ 11.19
Explained variance	27.5%	20.6%	14.3%	12.1%	Σ 74.6%

Factors are shown in bold.

In total 74.6% of the total variance could be explained with four extracted dimensions. Following the NTBV domain names, we labeled the extracted dimensions using the meta key-terms Vigilance, Verbal Memory, Executive functions and Verbal fluency.

The subsequent comparison of the four factor scores between the PD treatment subgroups using a Welch t-test is shown below in Table 4. The direction of the NTBV z-standardized factor scores indicates significantly better performance of DBS patients.

**Table 4.** Key values (*M*± *SD*; *Mdn*, IQR) of z-factor scores according to the extracted dimensions taking the two treatment subgroups into account (N=212).

NTBV dimension	Treatment	n	*M* ± *SD*	*p*-value. Effect size *d*
F1 Vigilance	BMT	134	-.205 ± 1.053	<.001^**^
	DBS	78	+.352 ± .784	-0.58
F2 Verbal memory	BMT	134	-.179 ± 1.066	<.001^**^
	DBS	78	+.307 ± .785	-0.50
F3 Executive functions	BMT	134	-.121 ± .955	<.023^*^
	DBS	78	+.208 ± 1.040	-0.33
F4 Verbal fluency	BMT	134	-.149 ± 1.067	<.002^**^
	DBS	78	+.257 ± .809	-0.41

^**^p ≤ .001.

^*^p ≤ .05; *d* = effect size Cohen’s Δ.

**Figure 3.**
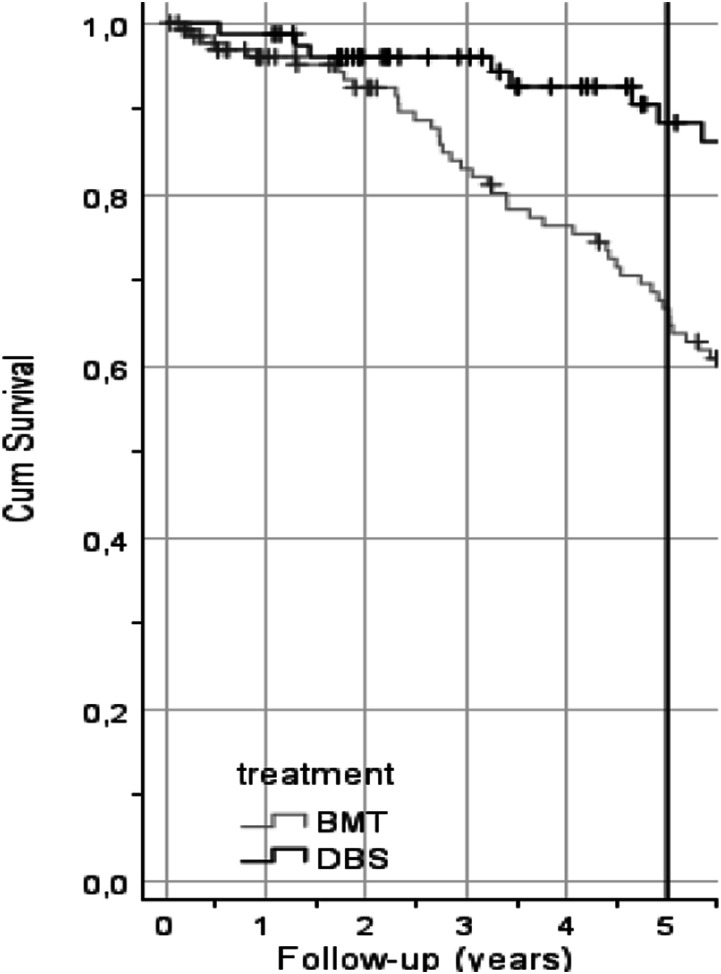
Kaplan Meier curves of the 5-year survival comparing PD treatment subgroup.

The KM 5-year-survival functions for the two treatment subgroups revealed a 5-year-survival rates of 66.7% for BMT, 88.4% for DBS and 74.4% for the entire study population. Survival probabilities for different follow-up periods are presented in table 4. The log-rank procedure revealed a significant difference *p* < .001 between the two subgroups with a higher cumulative survival likelihood for the DBS patients. The KM-functions regarding the two subgroups are presented in Figure 3. KM-survival likelihood regarding the follow-up period considering the two PD treatment subgroups is shown in (Table 5).

**Table 5.** KM-survival likelihood regarding the follow-up period considering the two PD treatment subgroups.

PD subgroup	n	6 month	1 year	2 years	3 years	5 years
BMT	134	97.7%	96.1%	92.5%	83.0%	66.7%
DBS	78	100%	98.7%	96.0%	96.0%	88.4%
Total	212	98.6%	97.1%	93.8%	87.9%	74.4%

In the first block of the Cox-proportional hazards model, represented in Table 6, cholesterol parameters could not be identified as significant risk factors *p* > .577. In the final block, with alle the covariates added into the model, the covariate age was identified as a significant risk factor *p* < .001 while the covariate sex was identified as a tendential risk factor *p* < 0.10. The other covariates including cholesterol parameters, educational years, GDS-15 score, NTBV factor scores and PD treatment revealed no significant risk regarding hazard ratios (HR) for the mortality of PD patients.

**Table 6.** Coefficients of predictors in the Cox-model for the mortality criterion considering PD treatment subgroups (n =174, cases with complete documented protocols).

Block	Predictor	*B*	*SE*	Wald (χ^2^), df=1	*p*-value	HR Exp(*B*)	95%-CI HR Exp(*B*)	
							LB	UB
First	TC	.005	.014	.104	.747	1.005	.977	1.032
	LDL	-.006	.011	.310	.577	.994	.972	1.016
	HDL	-.015	.026	.304	.581	.986	.936	1.038
	TC/HDL	-.073	.394	.034	.854	.930	.430	2.011
Final	TC	-.014	.017	.681	.409	.989	.955	1.019
	LDL	.008	.013	.370	.543	1.008	.983	1.034
	HDL	.002	.031	.466	.495	1.021	.961	1.085
	TC/HDL	.166	.443	.141	.707	1.181	.496	2.811
	age (years)	.081	.019	18.376	<.001^**^	1.084	1.045	1.125
	sex	-.532	.297	3.218	.073°	.587	.328	1.051
	school years	.005	.033	.026	.872	1.005	.942	1.073
	GDS-15	.010	.041	.062	.803	1.010	.932	1.094
	F1 Vigilance	-.181	.142	1.620	.203	.835	.632	1.102
	F2 Verb. memory	.093	.138	.450	.502	1.097	.837	1.438
	F3 Exec. function	-.137	.144	.912	.340	.872	.657	1.156
	F4 Verbal fluency	.106	.137	.468	.438	1.112	.850	1.454
	PD treatment	-.256	.345	.553	.457	.774	.394	1.521

^**^p ≤ .001.

^*^p ≤ .05, °p ≤ .10.

## Discussion

This study examined whether peripheral blood cholesterol parameters are associated with 5-year all-cause mortality in PD patients treated with BMT or DBS. Additionally, survival rates were analyzed by comparing the survival curves of both treatment groups using the Kaplan-Meier method and a log-rank test. To our knowledge, few studies examined the association between peripheral cholesterol and 5-year all-cause mortality. While a previous work from our center with an overlapping cohort examined the influence of depressive symptoms^15^ this study focused on cholesterol parameters this providing additional prognostic information.

A significantly higher number of male patients was observed when looking at the whole study population confirming the known higher prevalence of PD in men.^31^ Comparing the age of the two subgroups we found a significant difference with BMT patients being older than the DBS subgroups. This might also explain the observed significant difference in disease duration between the two subgroups. The median age of 60.8 years for DBS patients is in line with a previous study by Weaver et al. where DBS patients were also younger with an age of around 55 years.^32^

Neuropsychological testing revealed that patients in the DBS group performed significantly better across multiple cognitive domains (vigilance, memory, executive function, verbal fluency), while also exhibiting lower depressive symptom scores. These findings are in line with a previous study which also found DBS patients to have better depression scores and cognitive performance.^33^ These results could reflect the more rigorous selection criteria for DBS candidacy, which typically exclude patients with advanced cognitive impairment and uncontrolled psychiatric comorbidities.

Regarding the survival probabilities of the study population, the overall 5-year survival was 77.4%. Comparing the individual subgroups with a log-rank procedure we found a significantly higher survival for the DBS subgroups. This is in line with previous studies who also found DBS patients to have a higher cumulative survival when compared to BMT patients.^34,35^ However, treatment type did not emerge as a significant predictor of mortality when adjusting for other variables in the Cox proportional hazards model, suggesting that other factors may contribute to the observed survival advantage. The yearly reduction of survival probability observed in this study is similar to a previous study where the reduction was determined to be about 5% every year.^36^

When comparing the cholesterol parameters of the two subgroups we only found a significant difference for the TC/HDL-ratio with DBS patients having a slightly worse ratio than BMT patients. Taking a closer look at the total cholesterol levels of the study population, we found that TC levels tended to increase for the BMT as well as the DBS subgroup. This finding is in contrast to a previous study by Wilson et al. where it was observed that TC tends to decrease with age.^37^ This difference might be explained by a possible confounder like statin use which has not been accounted for in this study. Despite earlier studies suggestion a protective effect of higher cholesterol against the development of PD^9,38^ and a previous study by Yoon et al. suggesting a protective effect of higher cholesterol levels on PD mortality,^39^ our findings did not confirm a statistically significant influence of total cholesterol, LDL, HDL, or TC/HDL ratio on the mortality in PD patients giving it neither a protective effect nor a risk increase. These discrepancies may in part be explained by the fact that brain cholesterol homeostasis is largely regulated independently of the peripheral cholesterol parameters tested in this study.^40,41^ Peripheral cholesterol levels may be too weakly associated with PD mortality. A potentially more relevant biomarker might be CSF levels of 24-S-HC, which has been linked to PD disease progression.^7,8^ Furthermore, a single cross-sectional measurement of cholesterol parameters might not represent long-term lipid exposure and changes associated with disease progression or mortality. A recent study has associated statin use with lower all-cause mortality in PD patients.^42^ Statin exposure or metabolic factors my have influenced the results but these variables were not available in our dataset. Only the covariate age emerged as a significant predictor of mortality, consistent with the literature on both PD and general aging populations. The observed trend toward male sex as a risk factor (HR = 1.748, p < .10) suggests a possible influence that may warrant further investigation in larger samples to clarify its influence. These findings are however consistent with results from studies on Alzheimer’s disease (AD), mild cognitive impairment (MCI), and subjective cognitive decline, where higher age and male sex were also identified as significant risk factors for mortality^12,13^ and a study by Gerschmann and Lehrner (2024) which also found depressive symptoms to be a non-significant risk factor for mortality.^14^ In contrast to findings in AD^11–14^ neurocognitive performance was not a significant predictor of mortality in our PD cohort. This result should be interpreted with caution. In AD cognitive decline is a core clinical feature of the disease which is closely linked to disease severity, additionally greater disease severity has been linked with higher mortality in AD patients.^43^ Although PD is predominantly characterized by its motor symptoms which appear at earlier stages, cognitive impairment and dementia also become increasingly common in later stages of the disease and have been associated with reduced survival and increased mortality in previous studies.^44,45^ This difference might be explained by an introduced selection bias with severe cognitive impairment being an exclusion criterion for both treatment groups and the DBS group in particular which underwent strict selection procedures prior to the operation excluding individuals with cognitive impairment. Additionally, the follow-up interval of our study may have been too short to capture any effects of cognitive decline.

### Limitations

This study has several limitations. Due to this study being a retrospective data analysis mistakes during data collection and data transformation, cannot be ruled out. The sample size of N = 212, missing parameters for some of the neuropsychological test results and the monocentric approach could have led to lower interpretability value of the results of this study. Exclusion of patients with missing cholesterol parameters could have introduced a selection bias. Cholesterol levels were based on a cross-sectional survey, meaning that changes over time could not be assessed. Comorbidities such as cerebrovascular disease status, patient diet, nutritional status, body-mass index and statin which all pose possible influences on the cholesterol parameters have not been collected for this study and could not be adjusted for in the analysis. The lack of cause-specific mortality data also poses a limitation for this study as PD-related deaths cannot be distinguished from non-PD-related deaths.

The generalizability of our results is limited by the mono centric design and the specifically selected sample from the outpatient clinic of a university hospital. In particular, patients undergoing DBS are subjected to strict selection criteria and may therefore not be representative of the broader PD patient’s population. Additionally, the exclusion of cognitively impaired patients and those with cholesterol measurements outside of the defined range might further limit external validity. Application of our results to generalized PD patient populations should be done only with caution.

## Conclusion

In conclusion, the results of this study revealed that cholesterol parameters including TC, LDL, HDL and TC/HDL may have no substantial influence on the mortality of PD patients. In the Cox-regression model only the covariate age could be identified as a significant risk factor and male sex as a tendential risk factor. No significant differences in lipid profiles could be found between the treatment groups except for a higher TC/HDL-ratio in the BMT subgroup. Further studies are needed to understand the complex relationship between cholesterol and Parkinson’s disease.

Further studies should include a balanced sample size and, where feasible, propensity score matching to keep the influence of confounding variables largely constant. They should also account for statin use, ideally including dosage and duration of the treatment, to better account for medication effects on the cholesterol levels.

## Supplemental material

Supplemental material - The influence of cholesterol on the 5-year all-cause mortality of Parkinson patients with or without deep brain stimulation Supplemental material for The influence of cholesterol on the 5-year all-cause mortality of Parkinson patients with or without deep brain stimulation vy Gabriel C. Roth, Marco Treven, Christof Brücke and Johann Lehrner in Science Progress.

## Data Availability

The data can be obtained within reasonable request.
