# Supplementary material for: The influence of cholesterol on the 5-year all-cause mortality of Parkinson patients with or without deep brain stimulation
Source: Sci Prog. 2026 Jul 22;109(3):00368504261463435. doi: 10.1177/00368504261463435 (PMC13392330; doi:10.1177/00368504261463435)
Supplement: Supplemental material - The influence of cholesterol on the 5-year all-cause mortality of Parkinson patients with or without deep brain stimulation [file sj-pdf-1-sci-10.1177_00368504261463435.pdf]

ethik kommission
Medizinische Universität Wien

Borschkegasse 8b/6
1090 Wien, Österreich
T +43(0)1 404 00-21470, 22440
F +43(0)1 404 00-16900
ethik-kom@meduniwien.ac.at
http://ethikkommission.meduniwien.ac.at/

Votum

EK Nr: 2318/2024
Projekttitel: Der Einfluss von Cholesterin auf die Fünf-Jahres-Mortalität von Parkinson Patient/innen mit oder ohne tiefer Hirnstamm-Stimulation - eine retrospektive explorative Studie von 419 Patienten
Antragsteller/in: Herr Gabriel Christopher Roth
Institution: Medizinische Universität Wien
Sponsor: Medizinische Universität Wien

Art des Projektes:

- **Retrospektive Datenauswertung**
- **Diplomarbeit**

Teilnehmende Prüfzentren:

Ethik-Kommission	Prüfzentrum	Prüfärztin/arzt
Ethikkommission der Medizinischen Universität Wien	Univ. Klinik für Neurologie, Medizinische Universität Wien	Herr Assoc. Prof. Priv.-Doz.Mag.Dr. Johann Lehrner

Die Stellungnahme der Ethik-Kommission erfolgt aufgrund folgender eingereichter Unterlagen:

Lebenslauf (CV)

Name	Version	Datum
Lebenlauf_Lehrner_Juni24	1	03.06.2024

Sonstige

Name	Version	Datum
Verpflichtungserklaerung_Roth_V1	1	10.11.2024
Johann Lehrner Publikationsliste Juni 2023_PDF	1	11.11.2024
Ethikantrag_Roth_Unterschrieben_V1	1	21.11.2024

Studienprotokoll (Prüfplan)

Name	Version	Datum
Projektplan_Roth_V1.0	1.0	15.11.2024

Die Kommission fasst folgenden Beschluss (mit X markiert):

X	Es besteht kein Einwand gegen die Durchführung der Studie.<br><br>ACHTUNG: Unter Berücksichtigung der "ICH-Guideline for Good Clinical Practice" gilt dieser Beschluss ein Jahr ab Datum der Ausstellung. Gegebenenfalls hat der Antragsteller eine Verlängerung der Gültigkeit rechtzeitig zu beantragen.

Ergänzende Kommentare der Sitzung am 10.12.2024:

Hinweis:
Die Ethikkommission verweist auf die allenfalls erforderliche Konsultation der Rechtsabteilung der MedUni Wien, der Datenclearingstelle der MedUni Wien, des:der Datenschutzbeauftragten der MedUni Wien bzw. des Datenschutzverantwortlichen des AKH sowie auch auf die verpflichtend einzuhaltenden GSP Richtlinien der MedUni Wien und die Vorgaben des Handbuchs für Drittmittelprojekte der MedUni Wien.
Weitere Informationen finden sich unter https://ethikkommission.meduniwien.ac.at/service/weitere-informationen/

Die aktuelle Mitgliederliste der Ethik-Kommission ist unter folgender Adresse abrufbar:
http://ethikkommission.meduniwien.ac.at/ethik-kommission/mitglieder/

Mitglieder der Ethik-Kommission, die für diesen Tagesordnungspunkt als befangen anzusehen waren und daher laut Geschäftsordnung an der Entscheidungsfindung/Abstimmung nicht teilgenommen haben: Herr Johann Lehrner

Dieses Dokument ist für berechtigte Benutzer/innen in digitaler Form unter folgender Adresse abrufbar:
https://ekmeduniwien.at/vote/30842/download/

**Unterzeichner**	Dr. Jürgen Zezula
**Datum/Zeit-UTC**	2024-12-15T21:04:32Z
**Prüfinformation**	Informationen zur Prüfung der elektronischen Signatur finden Sie unter: https://www.signaturpruefung.gv.at
